# Comprehensive Analysis of the Expression and Prognosis for MCM4 in Uterine Corpus Endometrial Carcinoma

**DOI:** 10.3389/fgene.2022.890591

**Published:** 2022-06-03

**Authors:** Li-Peng Pei, Yun-Zheng Zhang, Guang-Ying Li, Jing-Li Sun

**Affiliations:** Department of Obstetrics and Gynecology, General Hospital of Northern Theater Command, Shenyang, China

**Keywords:** MCM4, uterine corpus endometrial carcinoma, prognosis, multi-omics analysis, RT-qPCR, immunohistochemistry

## Abstract

**Background:** Mini chromosome maintenance protein 4 (MCM4) belongs to the family of mini chromosome maintenance proteins (MCMs) that plays a crucial role in DNA replication and cell cycle regulation. Given that MCM4 has been reported to be aberrantly expressed in a variety of tumor tissues, and is strongly associated with poor patient prognosis, it has rarely been reported in uterine corpus endometrial carcinoma (UCEC).

**Methods:** We explored the role of MCM4 in UCEC through multi-omics analysis, including gene expression levels, survival prognosis, the biological function of interacting proteins, immune infiltration, and diagnostic value. Finally, these results were confirmed by biological experiments.

**Results:** MCM4 was highly expressed in various malignancies including UCEC compared to normal samples and was associated with poor prognosis in patients with UCEC [including OS (HR = 1.74, *p* = 0.009), PFI (HR = 1.73, *p* = 0.002), PFI (HR = 2.23, *p* = 0.003)]. In the Cox regression analysis, MCM4 was an independent prognostic biomarker. Further studies showed those interacting proteins of MCM4 were enriched in DNA repair and cell cycle. Moreover, high expression of MCM4 was accompanied by lower infiltration of immune cells such as Treg cells and B cells. The distribution of MCM4 expression in molecular and immune subtypes was significantly different (*p* < 0.05), with high expression in the copynumber high (CN_HIGH) molecular subtype and the IFN-gamma dominant (C2) immune subtype. RT-qPCR and immunohistochemistry results also showed that MCM4 expression was significantly upregulated in endometrial cancer tissues and negatively correlated with patient prognosis (*p* < 0.05). Subsequent biological experiments confirmed that MCM4 promoted cell growth and invasion and inhibited apoptosis *in vitro*.

**Conclusion:** Therefore, MCM4 could be a new potential biomarker for UCEC.

## Introduction

Uterine corpus endometrial carcinoma (UCEC), as the fourth most common gynecological tumor (Siegel et al., 2020; Winterhoff et al., 2020). The incidence is also increasing year by year, and the age of onset tends to be younger, posing a serious threat to women’s physical and mental health (Morice et al., 2016). 75% of patients with UCEC can be diagnosed at an early stage and have a 5-year survival rate of 85% (Legge et al., 2020). However, the clinical prognosis is often poor for patients with advanced and recurrent disease, as well as for patients with clinically aggressive histological types (e.g., high-grade endometrioid carcinoma, serous adenocarcinoma) (Sun et al., 2021). Exploring the pathogenesis of UCEC and identifying potential targets for early diagnosis is vital to improving the prognosis of UCEC patients.

The mini chromosome maintenance proteins (MCMs) are responsible for the maintenance of chromosome function and the initiation of DNA replication. The MCM family consists of eight main members that can function as multimers, of which mini chromosome maintenance proteins 4 (MCM4) is an important member of the MCM family (Issac et al., 2019; Uchiumi et al., 2020). MCM4 has ATPase activity, which is central to the unraveling of the DNA double helix unwinding enzyme and participates in the formation of replication forks (Giaginis et al., 2010). In recent years, the effect of MCM4 on tumors has been widely revealed, and it is highly expressed in tumor tissues of gastric, colorectal, breast and liver, and is associated with poor prognosis (Ahluwalia et al., 2019; Guo et al., 2020; Wang N et al., 2021; Zhou et al., 2021), which can be used as a reliable prognostic marker. Aberrant expression of MCM4 promotes cell proliferation and tumorigenesis and is closely associated with poor prognosis and clinicopathological features of patients (Xie et al., 2017). However, to date, the mechanism of action of MCM4 in endometrial cancer remains unclear.

Based on The Cancer Genome Atlas (TCGA) and Genotype-Tissue Expression (GTEx) databases, the expression levels of MCM4 in pan-cancer were first explored. To understand the mechanism of MCM4 in endometrial cancer, MCM4 expression levels and protein expression were compared between UCEC and normal paracancerous tissues, and the correlation between MCM4 expression levels and clinical characteristics was assessed. Then, 50 interacting proteins of MCM4 were screened through the Protein-protein interaction (PPI) database and functionally enriched to understand the possible involvement of MCM4 in biological functions. Subsequently, the correlation between the MCM4 and tumor microenvironment (TME) and immune cell infiltration was explored. The relationship between MCM4 expression and prognosis of endometrial cancer patients was analyzed based on clinical characteristics, and Cox regression and nomogram prognostic models were used to verify the clinical significance of MCM4 in UCEC. Finally, these results were confirmed by biological experiments *in vitro*, including quantitative real-iime polymerase chain reaction (qRT-PCR), immunohistochemistry, Cell Counting Kit-8 (CCK-8) assay, Transwell assay, and flow cytometry.

## Material and Methods

### Datasets and Preprocessing

Uniformly processed TCGA and GTEx pan-cancer RNA-seq data and relevant clinical characteristics data were downloaded from the UCSC website (https://xena.ucsc.edu/). First, the transcript levels of MCM4 in different cancers were analyzed through the Oncomine database (https://www.oncomine.org/resource/login.html). The TIMER 2.0 database (http://timer.cistrome.org/) allows systematic analysis of immune infiltration and exploration of gene expression and clinical prognosis in different cancer types (Li et al., 2020). The Oncogene Differential Expression Analysis module is used to analyze MCM4 gene expression differences in tumors and adjacent tissues. Finally, normal tissues from the GTEx database were added to further validate the differential levels of MCM4 transcription in different cancers, RNAseq data in TPM format of TCGA and GTEx processed uniformly by the Toil process (Vivian et al., 2017). Data were analyzed using a rank-sum test, “ggplot2” [version 3.3.3] R package was used for visualization, and *p* < 0.05 was considered statistically significant.

### Transcriptional Levels of MCM4 in Patients With UCEC

552 UCEC tissues and 35 adjacent non-tumor tissues from TCGA were extracted. The paired sample *t*-tests were performed to test for differences in MCM4 expression between 23 pairs of UCEC and normal tissues while analyzing the differences in MCM4 gene expression in tumor and adjacent tissues. In addition, the Human Protein Atlas (HPA) database (Pontén et al., 2008) (https://www.proteinatlas.org) at the protein level, and GSE17025 (Day et al., 2011) data were used for further MCM4 differential expression validation. Finally, differences in MCM4 expression levels were explored using Dunn’s test based on clinical characteristics grouped. *p* < 0.05 was considered statistically significant.

### Functional Enrichment Analysis

The 50 most highly associated MCM4 interacting proteins were obtained from the STRING database (Szklarczyk et al., 2021) (https://string-db.org/) and visualized using Cytoscape software. To better understand the functional significance of MCM4 in UCEC, a functional enrichment analysis of these 50 interacting proteins was performed using the “clusterProfiler” [version 3.14.3] and “org.Hs.eg.db” [version 3.10.0] R package, including Gene Ontology (GO) and Kyoto Encyclopedia of Genes and Genomes (KEGG) pathway analysis. Adjusted *p* values <0.05 and q values <0.05 were considered statistically significant.

### Tumor Microenvironment and Immune Cell Infiltration Analysis

The TME is the internal environment in which tumor cells survive and thrive. The “estimate” [version 1.0.13] R package was used to explore the differences in StromalScore, ImmuneScore, ESTIMATEScore between high and low MCM4 expression groups (Li et al., 2020). The immune infiltration analysis of MCM4 was performed by the “GSVA” [version 1.34.0] R package of the ssGSEA immuno-infiltration algorithm (Hänzelmann et al., 2013), including 24 infiltrating immune cells, namely activated DC (aDC), B cells, CD8 T cells, Cytotoxic cells, DC, Eosinophils, immature DC (iDC), Macrophages, Mast cells, Neutrophils, NK CD56bright cells, NK CD56dim cells, NK cells, Plasmacytoid DC (pDC), T cells, T helper cells, T central memory (Tcm), T effector memory (Tem), T follicular helper (Tfh), T gamma delta (Tgd), Th1 cells, Th17 cells, Th2 cells, and Treg.

In addition, the correlation between MCM4 expression and immune checkpoints in UCEC was further explored by the Wilcox test. The TISIDB database (http://cis.hku.hk/TISIDB/) integrates multiple types of data resources in tumor immunity (Ru et al., 2019), from which we explored the correlation between MCM4 expression and UCEC molecular subtypes (Copy Number High (CN_High), Copy Number Low (CN_Low), Microsatellite Instability (MSI), POLE) or immune subtypes (C1: wound healing, C2: IFN-gamma dominant, C3: inflammatory, C4: lymphocyte depleted, C6: TGF-b dominant). Spearman’s correlation analysis was used to assess the correlation between the variables and a *p*-value <0.05 was considered statistically significant.

### Prognostic Model Generation and Prediction

Kaplan-Meier curves were used to assess the relationship between MCM4 expression and UCEC prognosis [Overall survival (OS), Progression-free interval (PFI), Disease-specific survival (DSS)], the “survival” [version 3.2-10] R package is used for the statistical analysis of survival data and the “survminer” [version 0.4.9] R package for visualization. And the receiver operating characteristic (ROC) curves were used to assess the diagnostic value of MCM4 in UCEC. The area under the ROC curve generally ranges between 0.5 and 1. The closer the AUC is to 1, the better the diagnosis is. AUCs between 0.5 and 0.7 have low accuracy, AUCs between 0.7 and 0.9 have some accuracy, and AUCs above 0.9 have high accuracy (Mandrekar, 2010). Then, combined with clinical characteristics data, independent prognostic analysis was performed through univariate and multivariate Cox regression analysis. And a nomogram was then constructed to predict 1, 3, and 5-year survival rates in UCEC patients by the “rms” [version 6.2-0] and “survival” [version 3.2-10] R package, which was used to evaluate the prognostic value of the MCM4 in UCEC.

### Uterine Tissue Sample

We collected a total of 24 fresh frozen specimens each of cancerous and normal tissues (2019–2020), as well as pathological paraffin sections of 50 cancerous (2013–2015) and 20 normal (2021) tissues from the General Hospital of Northern Theater Command. The normal tissue specimens were obtained from patients who had undergone total hysterectomy or diagnostic curettage, none of whom had a confirmed endometrial lesion. The study was approved by the Ethics Committee of the General Hospital of Northern Theater Command and all patients gave their informed consent.

### Quantitative Real-Time Polymerase Chain Reaction

We used TRIzol reagent (Vazyme, Nanjing, China) to extract all RNA from endometrial cancer tissue and normal endometrial tissue. mRNAs were reverse transcribed into cDNAs using TransScript SuperMix (TransGen Biotech, Beijing, PRC). Green qPCR SuperMix (TransGen Biotech) was used as the fluorescent dye. The NCBI website was used to design the MCM4 primers, the positive chain sequence TGT​TTT​CCA​GCC​CTC​CCC​AAA​TG and the reverse chain sequence GAG​TGC​CGT​ATG​TCA​GTG​GTG​AAC, and to design the internal reference GAPDH primers, the positive chain sequence CAG​GAG​GCA​TTG​CTG​ATG​AT and the reverse chain sequence GAA​GGC​TGG​GGC​TCA​TTT. The qRT-PCR was performed using the ABI Prism 7500 with the following parameters: denaturation at 94°C (5 s), annealing and extension around 60°C (30 s), 40 cycles. The 2^−ΔΔCT^ method was used to calculate fold-changes (Livak and Schmittgen, 2001).

### Immunohistochemistry

We applied UltraSensitive SP kit (Maixin Biotech, Fuzhou, PRC) to perform the following immunohistochemical steps: After formalin-fixed paraffin sections had been pre-treated, de-formalinised and dehydrated, we applied a 3% concentration of hydrogen peroxide to the paraffin sections for 15 min to remove their endogenous peroxidase activity. We boil the paraffin sections in citrate buffer at pH = 6 for 8 min to expose the antigen and then cool to room temperature. Paraffin sections were reacted with MCM4 rabbit antibody (catalog number: A3018; 1:100 dilution; ABclonal, Wuhan, China) overnight at 4° and then rinsed with PBS buffer and reacted with the secondary antibody for 30 min at room temperature. Paraffin sections were autostained with DAB solution for 2 min and being counterstained, dehydrated and coverslipped were used to be viewed by fluorescent microscopy.

The immunoreactivity of MCM4 was scored based on both intensity of staining (negative = 0, weak = 1, moderate = 2, strong = 3) and percentage of immunoreactive tumor cells (<5% = 0, 5%–25% = 1, 25%–50% = 2, 50%–75% = 3, >75% = 4). The two scores are multiplied to give the final immunohistochemical score:0 scores (−), 1–4 scores (+),5–8 scores (++), and 9–12 scores (+++). The score of samples ≤4 was considered as low expression, and >4 was considered as high expression.

### Cell Line and Cell Culture

The human endometrial cancer cell line (Ishikawa) was obtained from the Institute of Biochemistry and Cell Biology, Chinese Academy of Sciences (Shanghai, China). Ishikawa cells were cultured with RPMI 1640 medium (Bioind, Kibbutz Beit Haemek, Israel) containing 10% fetal bovine serum (Bioind) and 1% penicillin-streptomycin (Invitrogen, Carlsbad, United States) under 5% CO_2_ at 37°C.

### Transfection of Cells

Short hairpin RNA (shRNA) targeting specific sequences in the human MCM4 mRNA was designed and synthesized by GenePharma (Target sequence: AAA​TGC​ATT​CTT​CAG​CTA​TCC​CT and AAA​TGT​TGG​CAT​AGA​TAT​TAC​TG; Jiangsu, PRC) to knockdown MCM4 expression, and a control shRNA that did not target MCM4 mRNA was synthesized as the negative control (si-NC). Normal Ishikawa cells were used as the untreated control. All cells were transfected using Lipofectamine 3000 according to the manufacturer’s instructions.

### Cell Proliferation Assay

Ishikawa cells were cultured in 96-well plates. Then 10 μl CCK-8 reagent (Cell Counting Kit-8, Dojindo, Japan) was added to each well. After incubation at 37°C with 5% CO_2_ for 3 h. The OD450 value of each well was measured using a microplate reader (Bio-Rad, Hercules, United States). Detection was performed at 0, 24, 48, and 72 h after treatment.

### Cell Invasion Assay (Transwell Assay)

Transwell filter inserts (8 μm pore size; Corning, NY, United States) were pre-coated with Matrigel (BD Pharmingen, NJ, United States) and washed with serum-free medium. Each upper chamber contained 2 × 104 starved cells resuspended with 200 μl serum-free medium, and each lower chamber was filled with medium containing 10% FBS. After incubation for 24 h, cells were washed three times and fixed with 4% poly-oxymethylene for 30 min, then stained with 0.1% crystal violet for 1 h. Three random fields were counted, and cell numbers were calculated by Image J (Version 1.8.0) software.

### Cell Apoptosis Detection by Flow Cytometry

After cell transfection, 1 × 10^6^ cells were collected from each group. These cells were washed once in PBS, and cells were stained with PE Annexin V and 7AAD using PE Annexin V Apoptosis Detection kit (BD Pharmingen, NJ, United States) at room temperature for 15 min. Then we used flow cytometry (BD FACSCalibur, NJ, United States) to evaluate the proportion of apoptotic cells.

### Statistical Analysis

The R package (version 3.6.3) and GraphPad Prism 7 (GraphPad Software, Inc., La Jolla, CA, United States) were used for statistical analysis. Wilcoxon rank sum test was used to assess the difference in MCM4 expression between normal and tumor tissues. Cancer patients were divided into high and low MCM4 expression subgroups according to the median of MCM4 expression. Survival prognosis was calculated using the Kaplan-Meier method and survival curves were compared using Cox regression. Spearman analysis was performed to assess the correlation between MCM4 expression levels and the level of immune cell infiltration and immune checkpoint-related genes. Each experiment was repeated three times independently. Data are presented as the mean ± standard deviation (SD). The *t*-test was used for two-way comparisons between groups, and the one-way ANOVA was used for multiple group comparisons. Differences were considered statistically significant at *p* < 0.05.

## Results

### Expression Levels of MCM4 in Patients With UCEC

**FIGURE 1:**
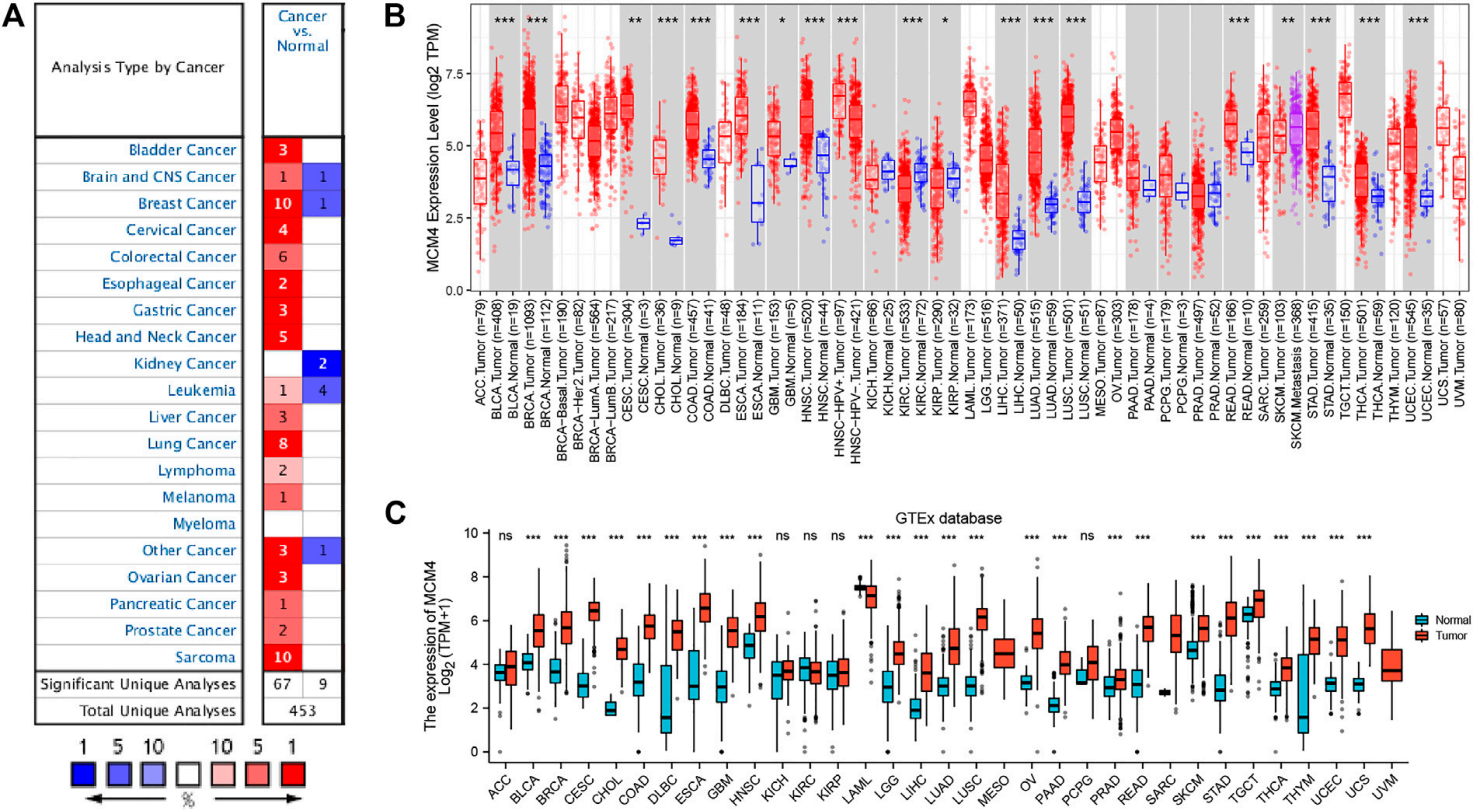
Expression level of MCM4 gene in Pan-Cancer. **(A)** The expression level of MCM4 gene in different types of Cancers (Oncomine database). **(B)** MCM4 expression in TCGA tumors and adjacent normal tissues (TIMER2.0 database). **(C)** MCM4 expression in TCGA tumors and normal tissues (GTEx database). *: *p*-value <0.05; **: *p*-value <0.01; ***: *p*-value <0.001. TCGA, the cancer genome Atlas; TIMER, tumor immune estimation resource; GTEx, genotype-tissue expression.

**FIGURE 2:**
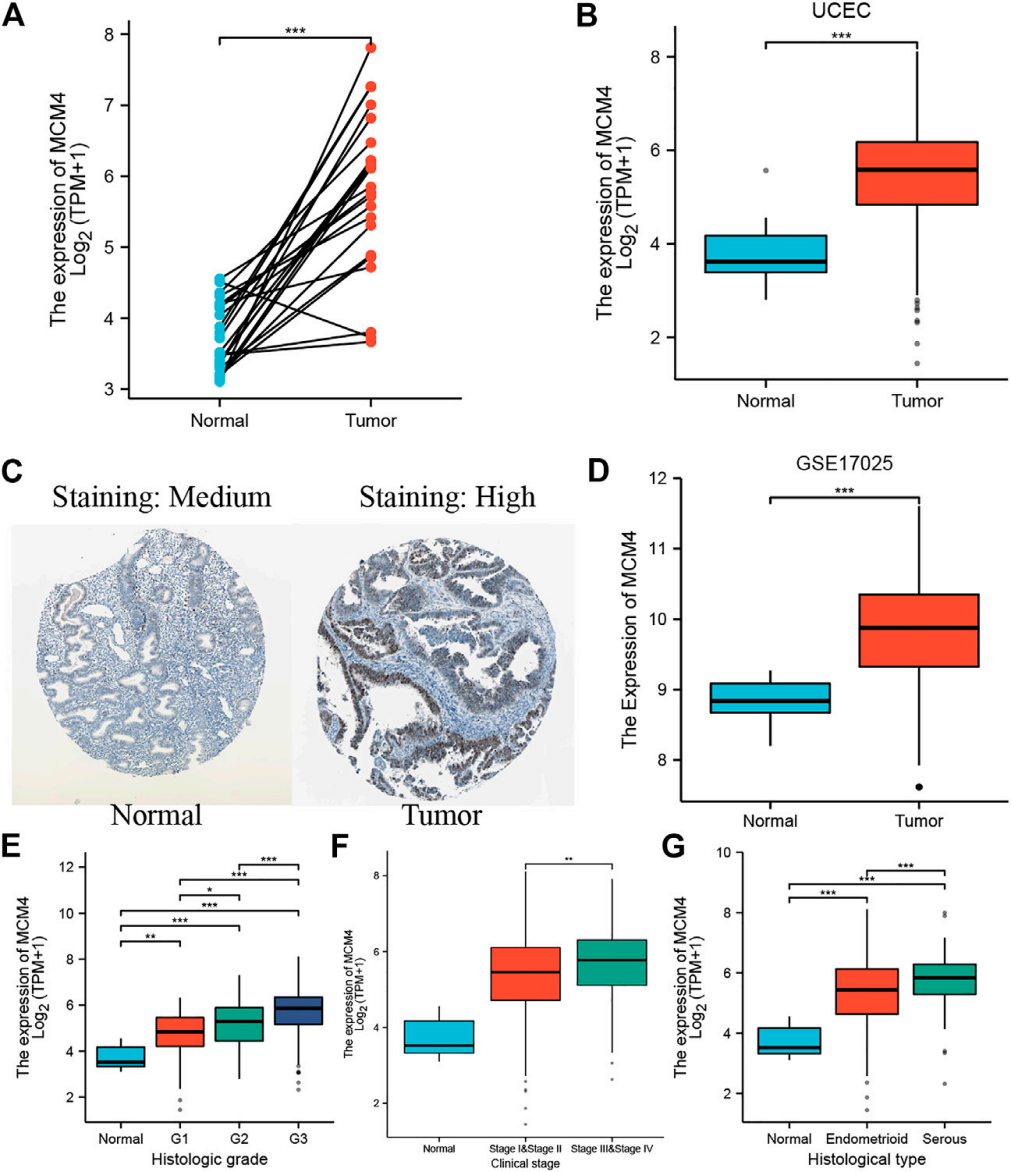
MCM4 expression in UCEC tissues. **(A)** The expression level of MCM4 in paired normal and UCEC samples; **(B)** Differential expression of MCM4 in UCEC tissues from TCGA; **(C)** The Expression of MCM4 in UCEC in protein level (The Human Protein Atlas). MCM4 was significantly upregulated in UCEC tissues compared with normal tissues; **(D)** Differential expression of MCM4 in UCEC tissues from GSE17025; **(E)** MCM4 expression associated with higher grade, **(F)** higher stage, and **(G)** histological type. *: p-value < 0.05; **: p-value < 0.01; ***: p-value < 0.001. UCEC, uterine corpus endometrial carcinoma; TCGA, the cancer genome Atlas.

Oncomine database compared the transcription level of MCM4 in cancer and normal samples. We found that the mRNA expression levels of MCM4 were significantly upregulated in a variety of cancer tissues, including bladder, breast, cervical, and ovarian cancers (Figure 1A). For TCGA tumors and adjacent normal tissues, MCM4 expression was significantly up-regulated in 15 cancer types, including Bladder Urothelial Carcinoma (BLCA), Breast invasive carcinoma (BRCA), Cervical squamous cell carcinoma and endocervical adenocarcinoma (CESC), Cholangiocarcinoma (CHOL), Colon adenocarcinoma (COAD), Esophageal (ESCA), Glioblastoma multiforme carcinoma (GBM), Head and Neck squamous cell carcinoma (HNSC), Liver hepatocellular carcinoma (LIHC), Lung adenocarcinoma (LUAD), Lung squamous cell carcinoma (LUSC), Rectum adenocarcinoma (READ), Stomach adenocarcinoma (STAD), Thyroid carcinoma (THCA), and UCEC, and downregulated in Kidney renal clear cell carcinoma (KIRC) and Kidney renal papillary cell carcinoma (KIRP) (Figure 1B). Furthermore, for the GTEx database as a normal tissue control, MCM4 expression was significantly upregulated in 24 cancer types, including BLCA, BRCA, CESC, CHOL, COAD, Lymphoid Neoplasm Diffuse Large B-cell Lymphoma (DLBC), ESCA, GBM, HNSC, Brain Lower Grade Glioma (LGG), LIHC, LUAD, LUSC, Ovarian serous cystadenocarcinoma (OV), Pancreatic adenocarcinoma (PAAD), Prostate adenocarcinoma (PRAD), READ, Skin Cutaneous Melanoma (SKCM), STAD, Testicular Germ Cell Tumors (TGCT), THCA, Thymoma (THYM), UCEC, and Uterine Carcinosarcoma (UCS), and downregulated in Acute Myeloid Leukemia (LAML) (Figure 1C). To further investigate the role of MCM4 in UCEC, we found that MCM4 was significantly upregulated in paired UCEC tissues from the TCGA database compared to adjacent normal tissues (Figure 2A). We obtained the same results when comparing all UCEC tissues with normal adjacent tissues (Figure 2B), and the same results were obtained for the expression pattern of MCM4 protein (Figure 2C). We further investigated MCM4 expression in the GSE17025 database and the results were consistent with the above results (Figure 2D). To investigate the relationship between MCM4 expression and clinical characteristics of UCEC, clinical characteristics were integrated, and the results showed that MCM4 expression was significantly correlated with histologic grade, clinical stage, histological type. MCM4 expression was significantly upregulated in the Grade 3 grouping (Figure 2E), Stage III + IV grouping (Figure 2F), and Serous grouping (Figure 2G).

### Functional Enrichment Analysis

**FIGURE 3:**
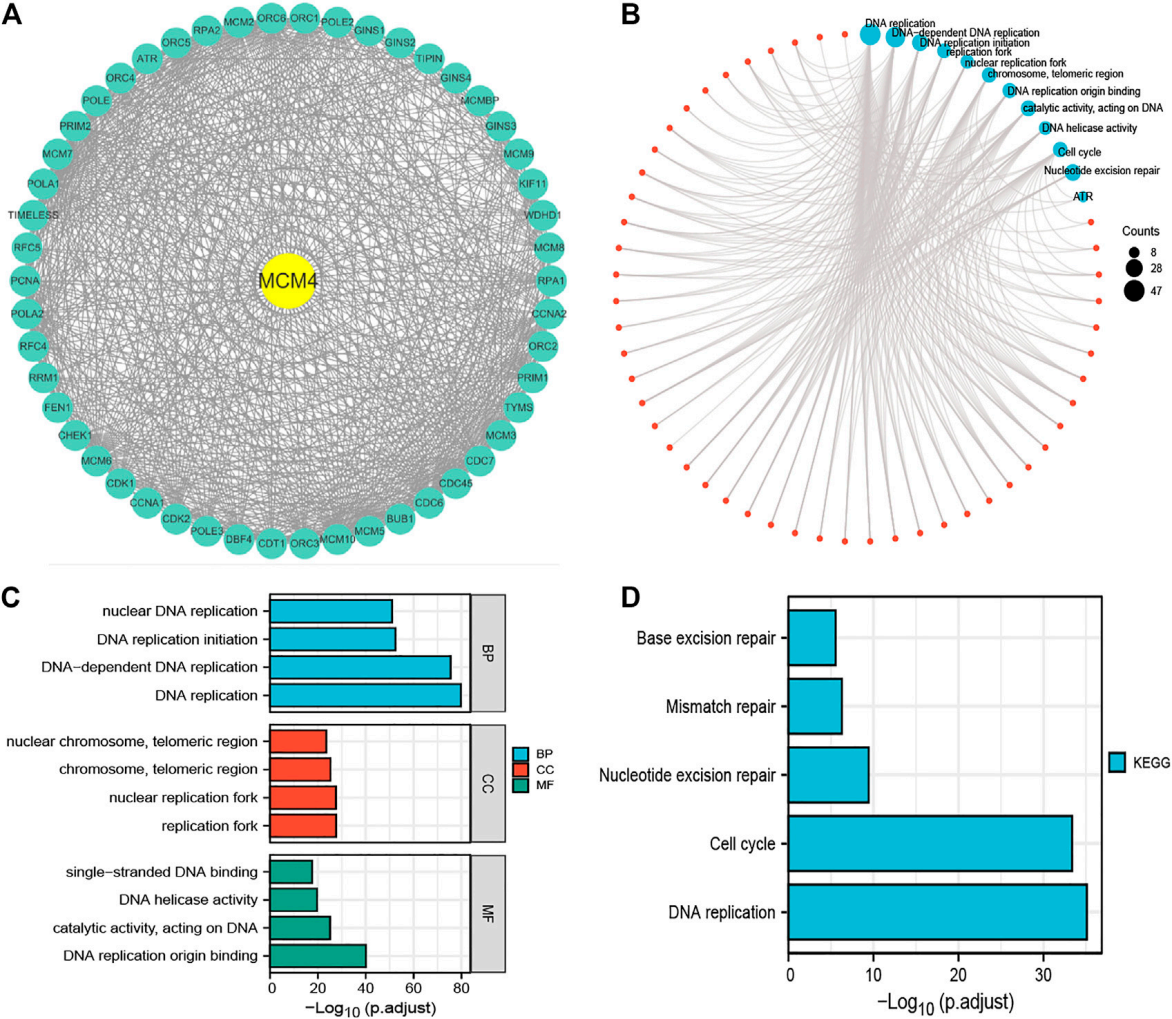
Functional enrichment analysis of 50 targeted binding proteins of MCM4. **(A)** PPI network; **(B)** Visual network of GO and KEGG analyses; **(C)** GO enrichment analysis with BP, CC, and MF; **(D)** KEGG pathway analysis. PPI, protein-protein interaction; GO, gene ontology; KEGG, kyoto encyclopedia of genes and genome; BP, biological process; CC, cellular composition; MF, molecular function.

50 interacting proteins of MCM4 were screened from the String database (Figure 3A), and functional enrichment analysis was performed on these target-binding proteins (Figure 3B). GO and KEGG enrichment results are summarized in Supplementary Table S1. In the BP, it is mainly enriched in DNA replication, DNA-dependent DNA replication, DNA replication initiation, and nuclear DNA replication. In the CC, they were mainly enriched in replication fork, nuclear replication fork, chromosome, telomeric region, nuclear chromosome, and telomeric region. In the MF, they were mainly enriched in DNA replication origin binding, catalytic activity, acting on DNA, DNA helicase activity, and single-stranded DNA binding (Figure 3C). In addition, the enriched KEGG pathway indicates that these proteins are mainly enriched in DNA replication, cell cycle, Nucleotide excision repair, Mismatch repair, and Base excision repair (Figure 3D).

### Correlation of TME and Immune Cell Infiltration With the MCM4 Expression

**FIGURE 4:**
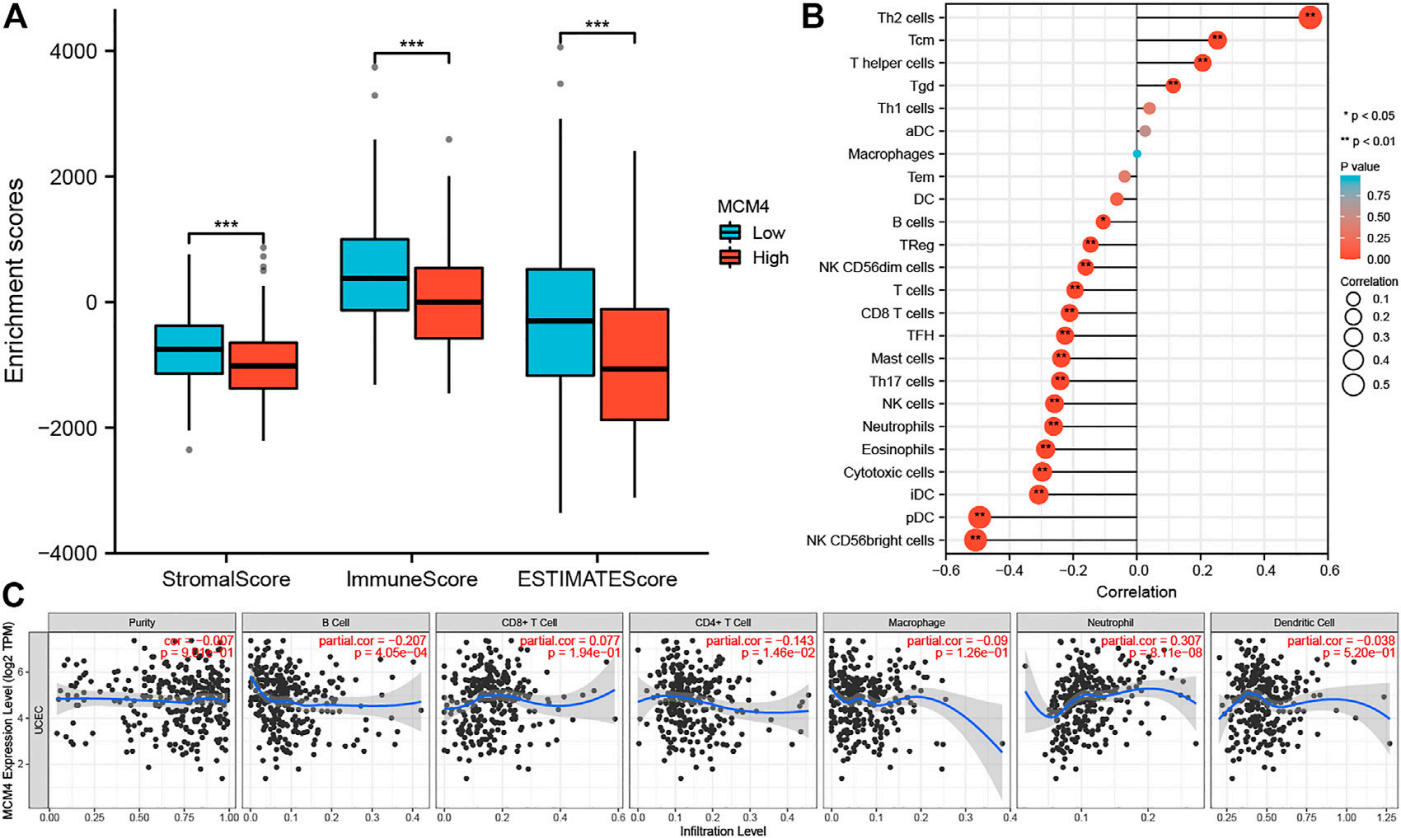
The relationship between the MCM4 gene and the tumor microenvironment and immune cell infiltration. **(A)** Patients with the high MCH4 expression had the lower stromal score, immune score, and estimate score. **(B)** The lollipop plot showed a significant positive correlation between MCM4 and four immune cell types and a significant negative correlation between MCM4 and 15 immune cell subpopulations. **(C)** MCM4 significantly correlated with B cell, CD4^+^ T cell, and Neutrophil infiltration in UCEC based on the Timer2.0 database. *: *p*-value <0.05; **: *p*-value <0.01; ***: *p*-value <0.001. TIMER, tumor immune estimation resource.

**FIGURE 5:**
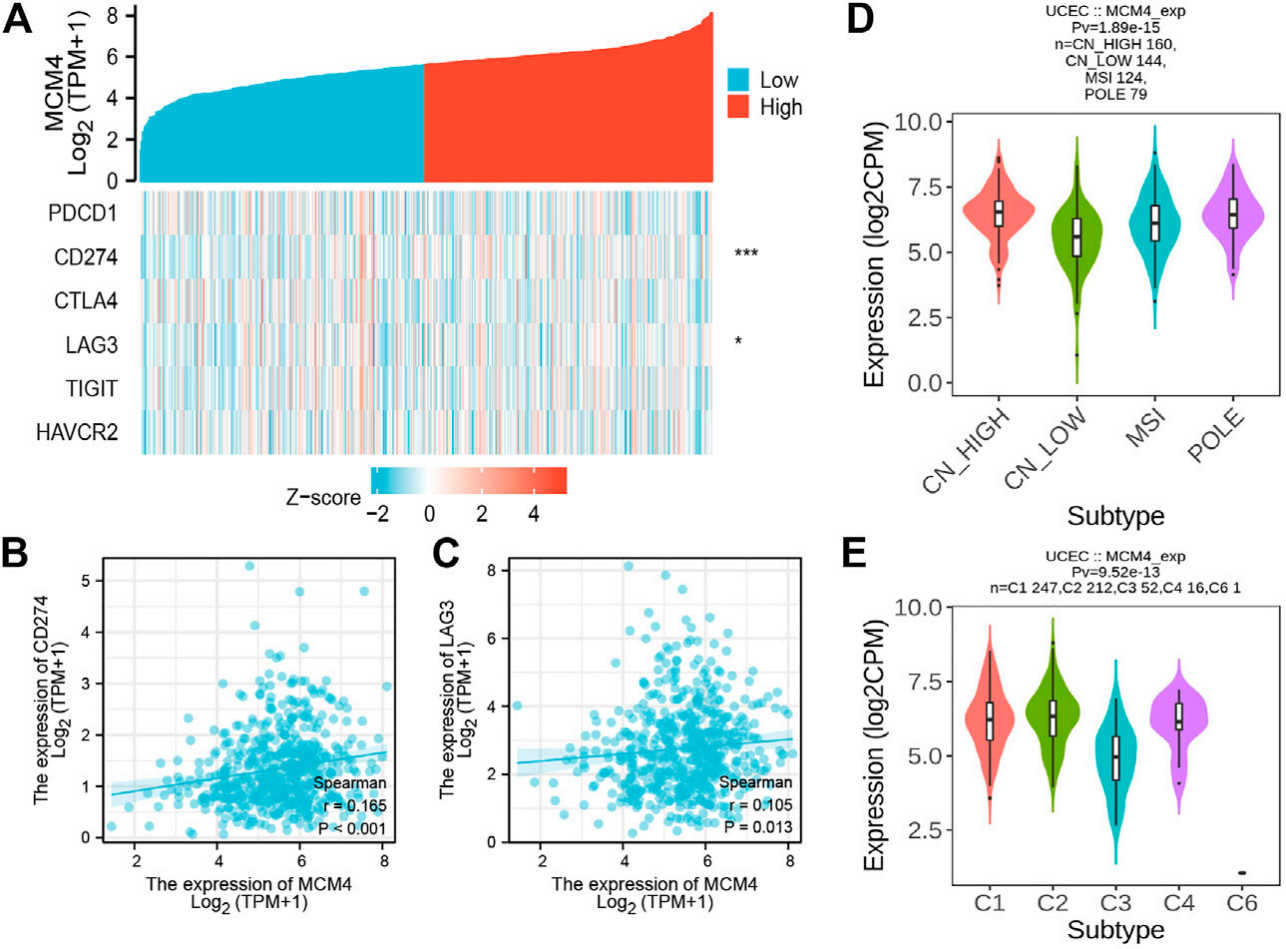
The relationship between the MCM4 gene and immune checkpoints, molecular subtypes, and immune subtypes. **(A)** Heatmap of immune checkpoints based on MCM4 expression. *: *p*-value <0.05; **: *p*-value <0.01; ***: *p*-value <0.001; **(B)** MCM4 expression was significantly and positively correlated with CD274 expression (R = 0.165, *p* < 0.001); **(C)** MCM4 expression was significantly and positively correlated with LAG3 expression (R = 0.105, *p* = 0.013); **(D)** Correlations between MCM4 expression and molecular subtypes; **(E)** Correlations between MCM4 expression and immune subtypes.

The occurrence and development of tumors are closely related to the surrounding environment. In the TME, tumors can influence their microenvironment by releasing cell signaling molecules that promote tumor angiogenesis and induce immune tolerance, while immune cells in the microenvironment can influence cancer cell growth and development (Korneev et al., 2017). We found that ESTIMATEScore, ImmuneScore, and StromalScore were all lower in patients with high MCM4 expression than in the low expression group (Figure 4A). Furthermore, MCM4 expression correlated with the level of infiltration of various immune cells (Figure 4B), with a significant positive correlation with infiltration of Th2 cells, Tgd, Tcm, T helper cells and a significant negative correlation with infiltration of B cells, TReg, NK CD56dim cells, T cells, CD8 T cells, TFH, Mast cells, Th17 cells, NK cells, Neutrophils, Eosinophils, Cytotoxic cells, iDC, pDC, NK CD56bright cells. The Timer2.0 database further verified that MCM4 expression was significantly negatively correlated with the level of infiltration of B cells (cor = −0.207, *p* < 0.001, Figure 4C). Given the importance of immunotherapy with immune checkpoint inhibitors, we further analyzed the correlation between MCM4 expression and immune checkpoints expression and showed that MCM4 expression was significantly positively correlated with CD274 and LAG3 (Figures 5A–C). We also observed that MCM4 expression was significantly associated with different molecular subtypes and different immune subtypes (Figures 5D,E).

### Prognostic Value of MCM4 in UCEC

**FIGURE 6:**
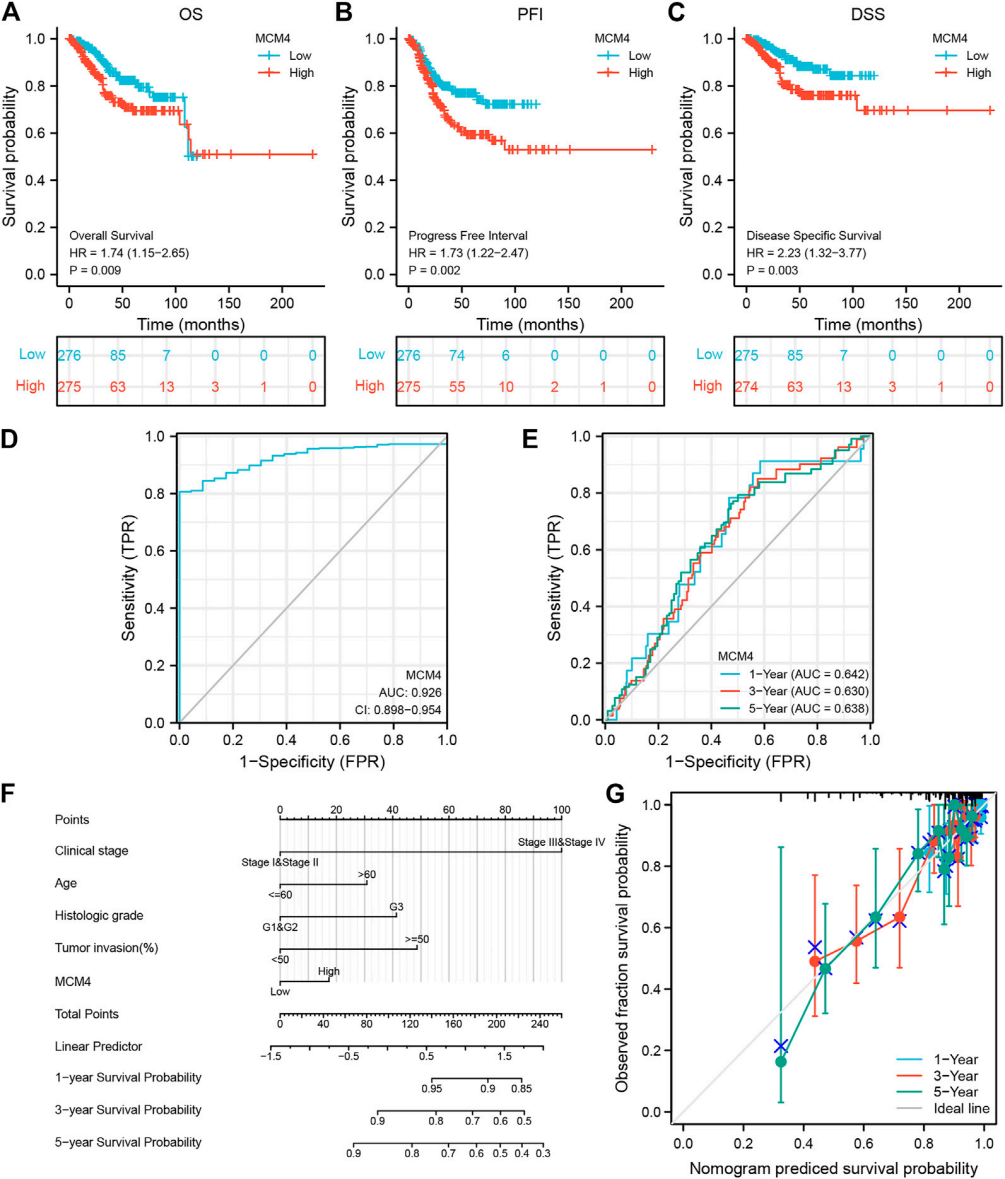
Prognostic model of MCM4 in UCEC. **(A)** Kaplan-Meier curves showed high expression of MCH4 was associated with poor OS. **(B)** Kaplan-Meier curves showed high expression of MCH4 was associated with poor PFI. **(C)** Kaplan-Meier curves showed high expression of MCH4 was associated with poor DSS. **(D)** The AUC values of the MCM4. **(E)** The AUC of the for the prediction of 1, 3, 5-year survival rate of UCEC. **(F)** Nomogram for both the MCM4 and clinical characteristics to predict 1, 3, 5-year survival rates. **(G)** Calibration plot for the nomogram predicting the probability of OS at 1, 3, and 5-year. UCEC, uterine corpus endometrial carcinoma. OS, overall survival. PFI, progression-free interval. DSS, disease-specific survival; AUC, area under the curve.

We further demonstrated the association between MCM4 and different clinical characteristics of UCEC and found that MCM4 expression was significantly associated with the Histologic grade (Supplementary Table S2). Cox regression results showed that high expression of MCM4 had a worse prognosis, including OS (*p* = 0.009, Figure 6A), PFI (*p* = 0.002, Figure 6B), and DSS (*p* = 0.003, Figure 6C). In addition, the potential value of MCM4 for diagnosing patients with UCEC was examined by ROC curve analysis with an AUC of 0.926 (Figure 6D), and it has good 1-year, 3-year, and 5-year predictive performance, with AUCs of 0.642, 0.63, and 0.638, respectively (Figure 6E). Moreover, the independent prognostic analysis showed that MCM4, Clinical stage, and Tumor invasion were independent prognostic factors for OS in UCEC patients (*p*-value <0.05, Table 1). The Nomogram for both the MCM4 and clinical characteristics is stable and accurate and can therefore be used to predict 1-, 3-, and 5-year survival in UCEC patients (Figure 6F). The predictive results of the calibration curve for the columnar plot of OS are consistent with the observations for all patients (Figure 6G). Overall, MCM4 has the potential as a biomarker.

**TABLE 1:** Independent prognostic analysis in UCEC.

Variables	Number	Univariate Cox regression analysis			Multivariate Cox regression analysis		
		HR	HR (95%CI)	*p*-value	HR	HR (95%CI)	*p*-value
Age		1.847	1.160–2.940	0.010	1.536	(0.912−2.586)	0.106
≤60	206						
>60	343						
BMI		0.967	0.636–1.470	0.876	—	—	—
≤30	211						
>30	307						
Hormone’s therapy		0.801	0.380–1.689	0.560	—	—	—
No	283						
Yes	45						
Stage		3.543	2.355–5.329	<0.001	4.019	2.433–6.639	<0.001
I + II	392						
III + IV	159						
Grade		3.281	1.907–5.643	<0.001	1.776	0.945–3.338	0.074
G1&G2	218						
G3	322						
Tumor invasion (%)		2.813	1.744–4.535	<0.001	1.967	1.157–3.346	0.013
<50	259						
≥50	214						
MCM4		1.742	1.147–2.645	0.009	1.893	1.076–3.340	0.034
Low	276						
High	275						

Abbreviations: UCEC, uterine corpus endometrial carcinoma; BMI, body mass index; HR, hazard ratio; CI, confidence interval.

### MCM4 Expression in Human UCEC Tissues

**FIGURE 7:**
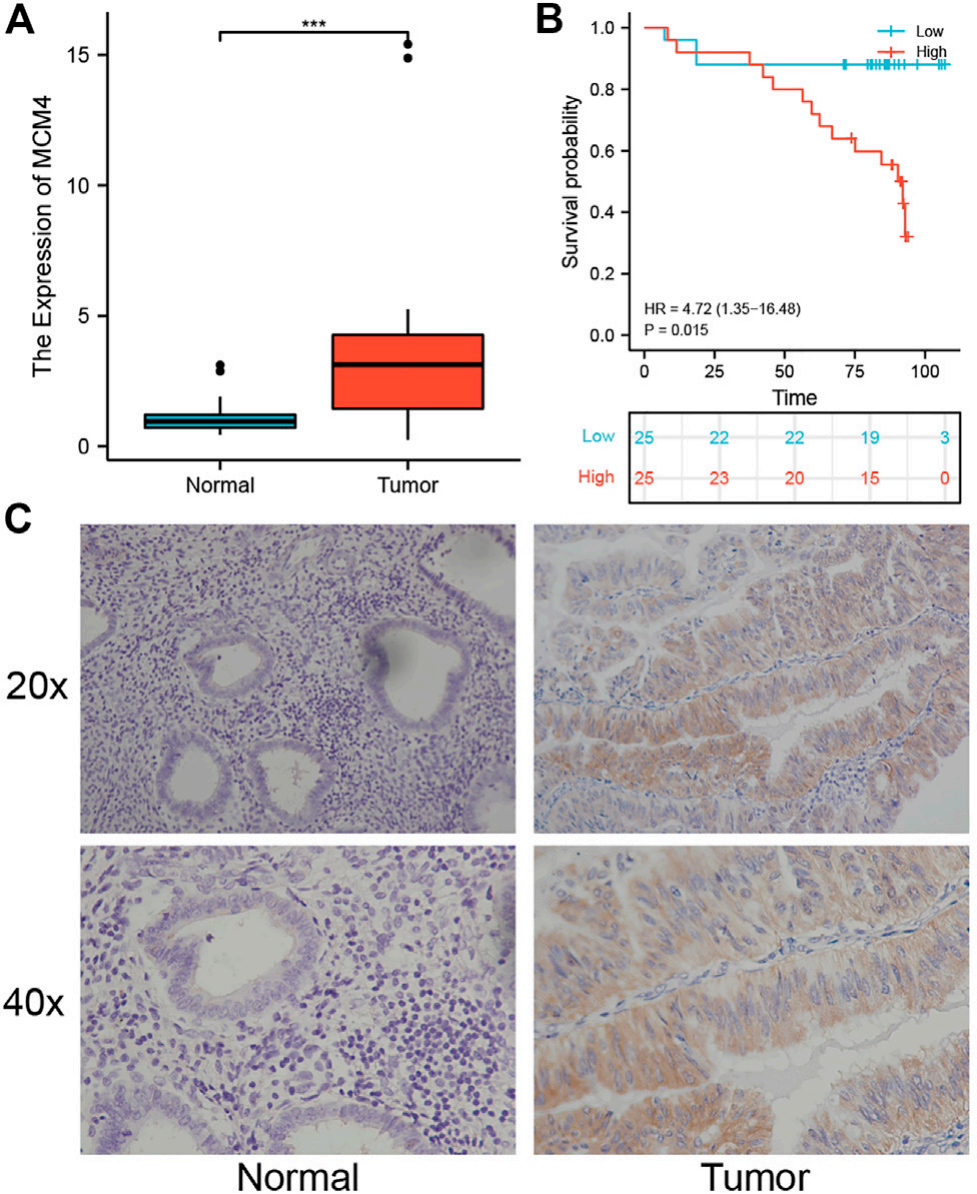
Expression and prognosis of MCM4 in human UCEC tissues. **(A)** Expression level of MCM4 in UCEC. MCM4 was significantly upregulated in human UCEC tissues compared with normal tissues. **(B)** Kaplan-Meier curves showed high expression of MCH4 was associated with poor OS in human UCEC tissues. **(C)** Immunohistochemical (IHC) staining of MCM4 expression (20× and 40×). UCEC, Uterine corpus endometrial carcinoma. OS, overall survival.

To further quantify the role of MCM4 in the development of UCEC, we collected fresh frozen specimens of 24 human UCEC tissues and normal tissues, and pathological paraffin sections of 50 cancerous and 20 normal tissues. The results showed that MCM4 was expressed at a higher level in cancerous tissues (Figure 7A), and the difference in staining intensity likewise indicated that MCM4 stained more significantly in cancerous tissues (Figure 7C). Survival prognosis analysis also showed that patients in the MCM4 high expression group had a poorer OS (Figure 7B). Details of the relationship between MCM4 expression and clinicopathological parameters in UCEC are shown in Table 2.

**TABLE 2:** Relationships between MCM4 expression in UCEC and clinicopathological parameters.

Characteristics MCM4	*n*	Low		High		High positive rate (%)	*p*-value
		(−)	(+)	(++)	(+++)		
Normal vs. tumor							<0.001
Normal tissue	20	17	3	0	0	0	
Tumor tissue	50	6	28	12	4	32.00	
FIGO stage							0.7635
I–II	29	3	16	8	2	34.48	
III–IV	21	3	12	4	2	28.57	
Age							0.0741
<65	44	6	26	9	3	31.82	
≥65	6	0	2	3	1	66.66	
Diferentiation							0.6994
Well-moderate	42	6	23	10	3	30.95	
Poor	8	0	5	2	1	37.50	
LN metastasis							0.7310
No	37	6	20	9	2	29.73	
Yes	13	0	8	3	2	38.46	

Abbreviation: UCEC, uterine corpus endometrial carcinoma.

### MCM4 Promotes Cell Growth and Invasion and Inhibits Apoptosis

**FIGURE 8:**
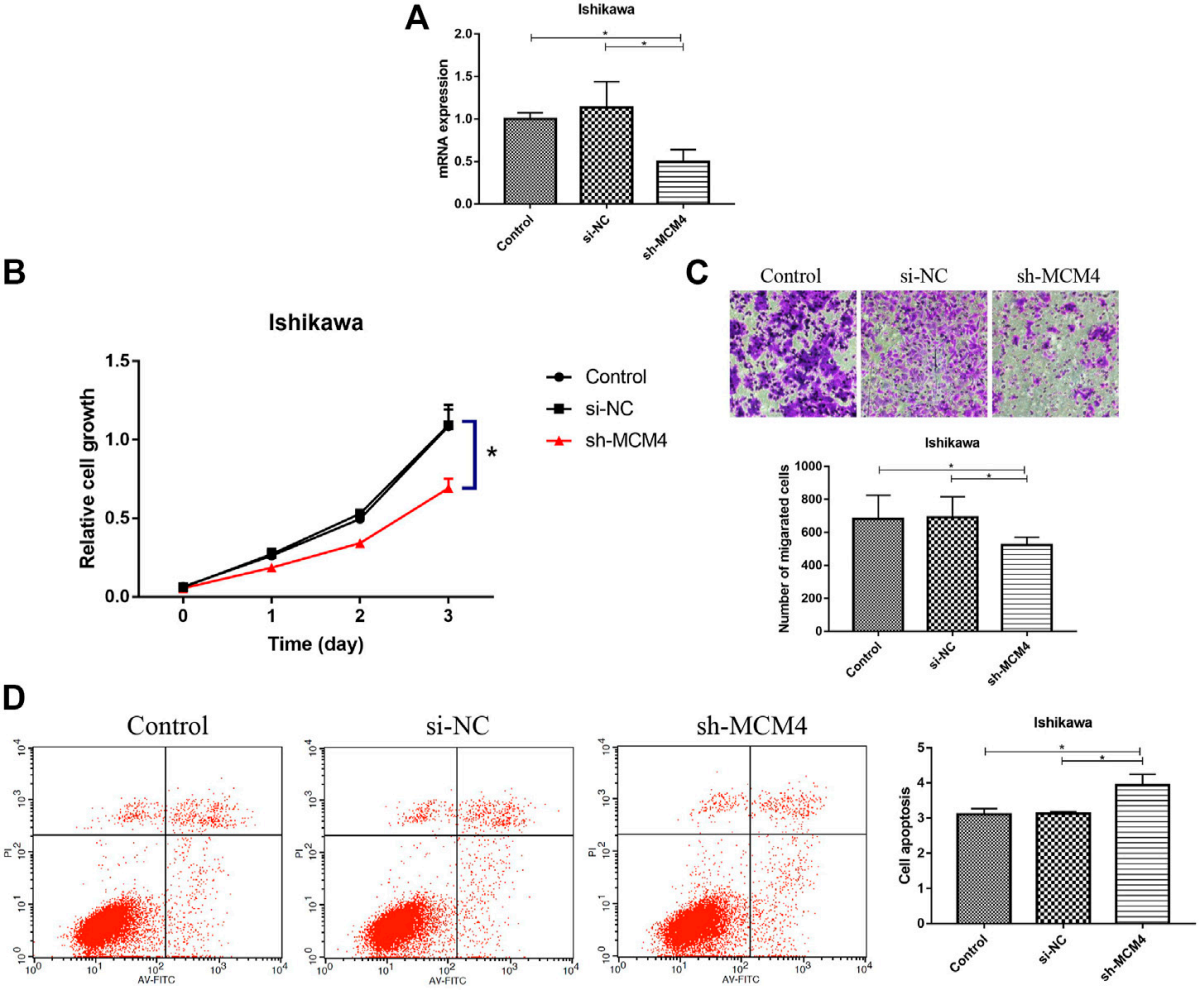
Knockdown of MCM4 inhibit cell proliferation, migration, and promotes apoptosis of Ishikawa cells. **(A)** MCM4 knockdown efficiency in Ishikawa cell lines were examined by qRT-PCR. **(B)** Effects of MCM4 silencing on proliferation of Ishikawa cells were monitored by the CCK-8 assay. **(C)** Effects of MCM4 silencing on invasion of Ishikawa cells were detected by the Transwell assay. **(D)** The apoptosis of Ishikawa cells after MCM4 knockdown was detected by flow cytometry. *: *p*-value <0.05. qRT-PCR, quantitative real-time polymerase chain reaction. CCK-8, cell counting kit-8.

We silenced the MCM4 expression in Ishikawa cells, and the knockdown efficiency is shown in Figure 8A. Compared with the Control and si-NC groups, MCM4 knockdown inhibited the growth (*p* < 0.05, Figure 8B) and invasion (*p* < 0.05, Figure 8C) of Ishikawa cells, and increased the apoptotic rate of Ishikawa cells (*p* < 0.05, Figure 8D). These results demonstrate that MCM4 promotes cell growth and invasion and inhibits apoptosis *in vitro*.

## Discussion

Endometrial cancer is often associated with the abnormal expression of genes and proteins (Czerwiński et al., 2021). In recent years, mortality rates have been on the rise (Wang J et al., 2021). Therefore, the search and discovery of endometrial cancer-related regulatory genes are important for the development of new therapeutic targets for UCEC. In recent years, more and more biomarkers of UCEC have been discovered based on bioinformatics. The impact of MCM4 on tumor development has been widely reported (Choy et al., 2016). As a cell cycle-related regulatory gene, MCM4 can regulate tumor progression through a variety of means. However, the regulatory mechanism between MCM4 and UCEC development is not fully understood. To further investigate the prognostic value of MCM4 regulation in UCEC, a multi-omics analysis revealed that MCM4 protein showed significantly high expression in endometrial cancer tissues and was closely associated with the clinicopathological characteristics and poor prognosis of patients.

Recent studies have shown that MCM4 is highly expressed in a variety of tumors including breast, ovarian, gastric, and colorectal cancers (Xie et al., 2017; Issac et al., 2019; Byun et al., 2020; Guo et al., 2020). However, to our knowledge, no studies are assessing the expression of MCM4 in pan-cancer. In this study, to assess the expression level of MCM4 in pan-cancer, we used the TCGA database and the GTEx database and found that it was significantly upregulated in 24 cancers, including BLCA, BRCA, CESC, CHOL, COAD, DLBC, ESCA, GBM, HNSC, LGG, LIHC, LUAD, LUSC, OV, PAAD, PRAD, READ, SKCM, STAD, TGCT, THCA, THYM, UCEC, and UCS, and downwards in LAML, KIRC, and KIRP. This finding suggests that MCM4 may act as a pro-oncogenic factor in most tumors, and may be involved in tumor formation and progression. In addition, we mainly analyzed the role of MCM4 in UCEC, confirmed that the expression and protein levels of MCM4 were significantly higher in endometrial cancer than in paracancerous tissues. RT-qPCR and immunohistochemical results also showed that the expression of MCM4 was significantly up-regulated in endometrial cancer tissues. A significant correlation between MCM4 expression level and histologic grade, clinical stage, and histological type was also identified.

Several studies have reported that abnormal expression of MCM4 is associated with poor prognosis in patients with a variety of tumors (Liu et al., 2021; Wu and Xi, 2021). Xu et al. (2021) found that patients with MCM4 high expression hepatocellular carcinoma had more advanced clinical stage and shorter survival time, and silencing MCM4 significantly inhibited the proliferation of hepatocellular carcinoma cells and the growth of hepatocellular carcinoma transplantation tumors. Overexpression of MCM4 is a potential prognostic marker for laryngeal squamous cell carcinoma, which is related to the poor prognosis of patients (Han et al., 2017). In addition, E2F2 induced upregulation of MCM4 expression in ovarian cancer and was significantly associated with the poor prognosis of patients (Xie et al., 2017). This is consistent with our findings that high MCM4 expression may lead to a worse prognosis for UCEC. Our *in vitro* experiments also validated that MCM4 promoted cancer cell growth and erosion and inhibited apoptosis in endometrial cancer cell lines, which correlated with poor prognosis in UCEC. Almost all UCEC risk stratification systems integrate clinical stage, grade and histologic type (de Boer et al., 2018). Furthermore, independent prognostic analysis suggests that MCM4 may have the ability to be an independent predictor of UCEC prognosis (univariate Cox: HR = 1.742, 95% CI = 1.147–2.645, *p* = 0.009; multivariate Cox: HR = 1.893, 95% CI = 1.076–3.340, *p* = 0.034). The MCM4 was combined with age, Clinical stage, Histologic grade, and Tumor invasion to construct a prognostic nomogram to obtain a more accurate prognostic prediction model. These results suggest that MCM4 is a potential molecular target for the diagnosis of UCEC.

MCM4 is a cell cycle-related regulatory gene that is important for the regulation of the cell cycle, cell division, and DNA replication (Champasa et al., 2019; Kim and Forsburg, 2020). Some researchers have demonstrated that phosphorylation of MCM4 by checkpoint kinases ATR and CDK2 can inhibit helicase activity, thereby inhibiting the DNA replication process (Hendrickson et al., 1996). This suggests that aberrant expression of MCM4 in tumor tissue could affect DNA replication and further affect the occurrence, development, and prognosis of tumors. Here, the interacting proteins of MCM4 were found to be enriched in DNA repair and cell cycle, which may be involved in endometrial carcinogenesis and maintenance. Further research is needed to confirm our results.

The tumor microenvironment includes immune cells and inflammation and plays a decisive role in tumor immunity (DeBerardinis, 2020). Studies have pointed out that immune function plays an important role in the development of endometrial cancer and that immune cell and their cytokines can influence the progression of endometrial cancer (Giatromanolaki et al., 2021). The immune system has a dual role in promoting and suppressing tumors. By analyzing the regulatory mechanisms between immune cells and tumor cells, it will be beneficial to subsequently optimize the prevention and treatment program for UCEC (Ikeda et al., 2017; Friedman et al., 2020). B cells are the main effector cells of humoral immunity.evidence that tumor-infiltrating B lymphocytes (TIB) can be observed in various solid tumors, inhibiting tumor progression by secreting immunoglobulins and killing cancer cells (Horikawa et al., 2011; Wang et al., 2019). Recently, a series of studies have shown that programmed cell death protein ligand-1 (PD-L1) plays an important role in various malignant tumors. High expression of PD-L1 plays a negative immune regulatory role by attenuating the host immune response to tumor cells and deactivating T lymphocytes to induce tumor immune evasion (Han et al., 2020). Additionally, McConechy et al., (2016) reported that the POLE and MSI molecular subtypes of endometrial cancer, typical of the high mutational load subtypes, produce a large number of tumor-specific neoantigens and tumor-infiltrating lymphocytes that participate in the active immune microenvironment, leading to overexpression of PD-1 and PD-L1. When activated T cells express PD-1, they bind to PD-L1 receptors on the surface of antigen-presenting cells and transmit negative regulatory signals to activated T cells, causing apoptosis or reduction of T cells, suppressing the function of T lymphocytes, triggering immune self-suppression, and ultimately playing an immune escape role (Niu et al., 2022). In the immune cell infiltration analysis, high expression of MCM4 was found to be associated with lower T cell infiltration and positively correlated with CD274 (PD-L1) expression. It may promote proliferation and growth of endometrial cancer cells by inhibiting anti-tumor immune cell activity and inducing immune tolerance or escape of cancer cells but remains to be further explored.

In summary, our study confirms the diagnostic and prognostic value of MCM4 in UCEC. However, there are some limitations to our study, and whether MCM4 directly or indirectly regulates the regulatory mechanisms of immune response needs further investigation.

## Data Availability

The original contributions presented in the study are included in the article/Supplementary Material, further inquiries can be directed to the corresponding author.
