# Supplementary material for: Comprehensive Analysis of the Expression and Prognosis for MCM4 in Uterine Corpus Endometrial Carcinoma
Source: Front Genet. 2022 Jun 3;13:890591. doi: 10.3389/fgene.2022.890591 (PMC9203964; doi:10.3389/fgene.2022.890591)
Supplement: Supplementary file 1 [file Table1.docx]

**Supplementary Table S1 Functional enrichment analysis of 50 targeted binding proteins of MCM4**

| ONTOLOGY | ID | | Description | | GeneRatio | | BgRatio | | pvalue | | p.adjust | | qvalue | |
| --- | --- | --- | --- | --- | --- | --- | --- | --- | --- | --- | --- | --- | --- | --- |
| BP | GO:0006260 | | DNA replication | | 47/51 | | 274/18670 | | 2.59e-83 | | 1.68e-80 | | 9.48e-81 | |
| BP | GO:0006261 | | DNA-dependent DNA replication | | 41/51 | | 153/18670 | | 9.77e-79 | | 3.17e-76 | | 1.79e-76 | |
| BP | GO:0006270 | | DNA replication initiation | | 24/51 | | 37/18670 | | 1.57e-55 | | 3.40e-53 | | 1.92e-53 | |
| BP | GO:0033260 | | nuclear DNA replication | | 26/51 | | 60/18670 | | 6.07e-54 | | 9.83e-52 | | 5.56e-52 | |
| CC | GO:0005657 | | replication fork | | 17/51 | | 70/19717 | | 3.72e-30 | | 2.38e-28 | | 1.06e-28 | |
| CC | GO:0043596 | | nuclear replication fork | | 15/51 | | 41/19717 | | 9.61e-30 | | 3.08e-28 | | 1.37e-28 | |
| CC | GO:0000781 | | chromosome, telomeric region | | 19/51 | | 161/19717 | | 2.76e-27 | | 5.89e-26 | | 2.62e-26 | |
| CC | GO:0000784 | | nuclear chromosome, telomeric region | | 17/51 | | 125/19717 | | 1.72e-25 | | 2.76e-24 | | 1.22e-24 | |
| MF | GO:0003688 | | DNA replication origin binding | | 18/51 | | 24/17697 | | 8.28e-43 | | 9.35e-41 | | 5.23e-41 | |
| MF | GO:0140097 | | catalytic activity, acting on DNA | | 21/51 | | 213/17697 | | 1.50e-27 | | 8.46e-26 | | 4.73e-26 | |
| MF | GO:0003678 | | DNA helicase activity | | 14/51 | | 81/17697 | | 6.13e-22 | | 2.31e-20 | | 1.29e-20 | |
| MF | GO:0003697 | | single-stranded DNA binding | | 14/51 | | 113/17697 | | 8.66e-20 | | 2.45e-18 | | 1.37e-18 | |
| KEGG | hsa03030 | DNA replication | | 19/38 | | 36/8076 | | 2.11e-37 | | 7.80e-36 | | 4.66e-36 | |  |
| KEGG | hsa04110 | Cell cycle | | 24/38 | | 124/8076 | | 2.30e-35 | | 4.26e-34 | | 2.55e-34 | |  |
| KEGG | hsa03420 | Nucleotide excision repair | | 8/38 | | 47/8076 | | 3.02e-11 | | 3.73e-10 | | 2.23e-10 | |  |
| KEGG | hsa03430 | Mismatch repair | | 5/38 | | 23/8076 | | 5.55e-08 | | 5.14e-07 | | 3.07e-07 | |  |

Abbreviation: GO, Gene Ontology; CC, Cellular Composition; BP, Biological Process; MF, Molecular Function; KEGG, Kyoto Encyclopedia of Genes and Genomes.
