# Supplementary material for: Comprehensive Analysis of the Expression and Prognosis for MCM4 in Uterine Corpus Endometrial Carcinoma
Source: Front Genet. 2022 Jun 3;13:890591. doi: 10.3389/fgene.2022.890591 (PMC9203964; doi:10.3389/fgene.2022.890591)
Supplement: Supplementary file 2 [file Table2.docx]

| Characteristic | Low expression of MCM4 | High expression of MCM4 | p |
| --- | --- | --- | --- |
| n | 276 | 276 |  |
| Age, n (%) |  |  | 0.605 |
| <=60 | 107 (19.5%) | 99 (18%) |  |
| >60 | 169 (30.8%) | 174 (31.7%) |  |
| BMI, n (%) |  |  | 0.652 |
| <=30 | 104 (20%) | 108 (20.8%) |  |
| >30 | 158 (30.4%) | 149 (28.7%) |  |
| Hormones therapy, n (%) |  |  | 0.297 |
| No | 149 (43.3%) | 148 (43%) |  |
| Yes | 28 (8.1%) | 19 (5.5%) |  |
| Clinical stage, n (%) |  |  | 0.070 |
| Stage I | 186 (33.7%) | 156 (28.3%) |  |
| Stage II | 23 (4.2%) | 28 (5.1%) |  |
| Stage III | 55 (10%) | 75 (13.6%) |  |
| Stage IV | 12 (2.2%) | 17 (3.1%) |  |
| Histologic grade, n (%) |  |  | < 0.001 |
| G1 | 77 (14.2%) | 21 (3.9%) |  |
| G2 | 77 (14.2%) | 43 (7.9%) |  |
| G3 | 117 (21.6%) | 206 (38.1%) |  |
| Tumor invasion(%), n (%) |  |  | 0.838 |
| <50 | 135 (28.5%) | 124 (26.2%) |  |
| >=50 | 115 (24.3%) | 100 (21.1%) |  |

**Supplementary Table S2 Clinical Characteristics of the UCEC Patients Based on TCGA**
